# Characterization of the conjugative modules of the pRAS4 plasmid of *Aeromonas salmonicida* reveals an environmental lineage

**DOI:** 10.3389/fmicb.2026.1855649

**Published:** 2026-07-06

**Authors:** Surya Prasad Dahal, Saurabh Dubey, Hetron M. Munang’andu, Henning Sørum

**Affiliations:** 1 Faculty of Biosciences and Aquaculture, Nord University, Bodø, Norway; 2 Department of Paraclinical Sciences, Faculty of Veterinary Medicine, Norwegian University of Life Sciences, Ås, Norway

**Keywords:** *Aeromonas salmonicida*, aquaculture, conjugative plasmid, environmental lineage, MobA/VirD2 relaxase, pRAS4, tetracycline resistance, type IV coupling protein

## Abstract

Conjugative plasmids of *Aeromonas salmonicida*, the causative agent of furunculosis in salmonids, have long been implicated in the dissemination of antimicrobial resistance in aquaculture. Among these, pRAS4 has remained the least characterized, with no complete published annotation and no molecular description of its conjugation machinery. In the present study, we report the complete sequence of pRAS4 from two *A. salmonicida* subsp. *salmonicida* strains, F878/91 and F830/91, isolated from Atlantic salmon (*Salmo salar* L.) showing characteristic signs of furunculosis on the Western coast of Norway in 1991. Mating experiments produced antibiotic-resistant transconjugants in *Escherichia coli* DH5α, and antimicrobial susceptibility testing of single-colony-purified transconjugants confirmed selective transfer of the tetracycline-resistance phenotype encoded by pRAS4. Domain based re-annotation using InterProScan identified two previously unassigned coding sequences as canonical components of the conjugation machinery: an 823-amino-acid coding sequence originally annotated as *traN* was reassigned as a TraG/VirD4-family type IV coupling protein (Pfam PF02534, *E* = 5.1 × 10^−99^), and a 366-amino-acid coding sequence originally labelled “hypothetical protein” was identified as a MobA/VirD2 family relaxase (Pfam PF03432, *E* = 4.7 × 10^−14^). Together with the canonical *virB1–virB11* mating pair formation cluster and the mobC accessory relaxosome gene, these findings establish that pRAS4 encodes a complete and functional MOB_P/MPF_T conjugation system. PlasmidFinder analysis returned no replicon match for pRAS4, and BLASTp of the RepA protein returned no close match for the canonical IncU plasmid RepAs (pRAS1, pAr-32, pRA3). The closest sequence relatives of the pRAS4 RepA, type IV coupling protein and relaxase are environmental plasmids of *Acidithiobacillus, Acidovorax, Microvirgula* and related β- and γ-proteobacteria, and the pRAS4 backbone shares an extensive synteny with the plasmids pYKCT010 and pYK0414 from uncultured bacteria of Japanese river sediments. These findings indicate that pRAS4 is a self-transmissible R-plasmid carrying a previously uncharacterized environmental plasmid backbone, and the tetracycline Tet A efflux determinant flanked by Tn*3* transposases represents a subsequent adaption under aquaculture associated antibiotic selection.

## 1 Introduction

Furunculosis also referred to as the “great red plague” of salmonids was first reported from brown trout (*Salmo trutta*) in Germany by Emmerich and Weibel (1894). By the early 1900s, it spread to several European countries and later to the USA and Canada by the mid-1930s (Bernoth, 1997). In Norway, it was first reported in 1964 from a rainbow trout (*Oncorhynchus mykiss*) farm after import of smolt from Denmark. In 1966 furunculosis was detected in wild Atlantic salmon in the nearby Numedalslågen river with varying degrees of disease in the river the following years. There was no occurrence of furunculosis in Norwegian aquaculture farms between 1970 and 1984. It resurfaced in mid-Norway in 1985 after import of infected smolt from Scotland (Johnsen and Jensen, 1994). The disease is caused by *Aeromonas salmonicida* and is currently causing high economic losses in aquaculture globally (Sørum and L'Abée-Lund, 2002; Vanden Bergh et al., 2013; Boily et al., 2019; Lim and Hong, 2020; Mancilla et al., 2025). Regulations regarding its control in continental European countries are focused on the control measures against the classical furunculosis etiological agent *A. salmonicida* subsp. *salmonicida* while control regulations in the USA, United Kingdom and most countries in the world are focused on the entire *A. salmonicida* covering both typical and atypical strains (Austin and Austin, 2016). *A. salmonicida* isolates encode multiple inherent plasmids with some additional laterally acquired plasmids carrying antimicrobial resistance (AMR) genes. Most *A. salmonicida* subsp. *salmonicida* isolates carry several small and one large inherent genomic plasmid (Bast et al., 1988; Sørum et al., 1993). The size varies from 1 to 6 kb for small plasmids while larger plasmids vary from 45 to 150 kb (Bast et al., 1988; Belland and Trust, 1989; Hänninen et al., 1995; Sørum et al., 2000; Boyd et al., 2003; Tanaka et al., 2017; Marcoux et al., 2023). Some studies have shown that large conjugative R-plasmids can be self-transferable while small plasmids are nonconjugative but may be mobilizable needing the help of large conjugative plasmids for transfer, collectively, leading to horizontal gene transfer (HGT) of AMR genes through plasmid conjugation (Sørum, 1998; Schmidt et al., 2001; Bruun et al., 2003). These revelations have led scientists from several countries engaged in intensive aquaculture like Chile (Valdes et al., 2015; Godoy et al., 2023), Japan (Aoki et al., 1983), Korea (Woo et al., 2025), Finland (Hänninen et al., 1995), Canada (Belland and Trust, 1989; Boyd et al., 2003; McIntosh et al., 2008; Vincent et al., 2014), USA (Adams et al., 1998), Denmark (Pedersen et al., 1996; Bartkova et al., 2017), France (Vincent et al., 2021), Scotland (Inglis et al., 1993), and Norway (L'Abée-Lund and Sørum, 2000, 2001; L’Abée-Lund and Sørum, 2002; Sørum et al., 2003) to investigate the potential role of *A. salmonicida* plasmids in the spread of AMR genes.

Conjugative plasmids are made of conserved backbone modules organized in a stable self-replicon that contain restricted gene clusters that mediate DNA transfer, maintenance and ensure inheritance stability after transfer into recipient cells (Frost et al., 2005; Waksman, 2019). A key module conserved in all conjugative plasmids of Gram-negative bacteria, is the mating pair formation (MPF) module that consists of gene clusters that form the type IV secretory system (T4SS) whose nanotubule works with the relaxosome processing machinery that orchestrates the unidirectional transfer of plasmid DNA from donor to transconjugants (Ilangovan et al., 2015; Waksman, 2019; Virolle et al., 2020). The MPF module also encodes coupling genes that facilitate contact of donors with recipient cells enabling the transfer (*tra*) genes to move plasmid ssDNA into recipient cells. The resolvase and replication modules carry genes like resolvase (*res*) that resolve the integration of plasmid ssDNA from donor into recipient cells (Crellin and Rood, 1997) and replicases, like *repA*, that initiate the replication of plasmid ssDNA into dsDNA in recipient cells (Díaz-López et al., 2003). The maintenance and inheritance stability module encodes genes, like *eexN*, that prevent deposition of excess plasmid DNA in recipient cells (Humbert et al., 2019) as well as genes that prevent degradation of plasmid DNA in recipient cells through antitoxin and anti-restriction functions (Feng et al., 1994; Schureck et al., 2019). Overall, the composition of gene clusters that form the backbone modules of conjugative plasmids is designed to ensure their efficient self-transfer, initiation of replication, integration and protection against adverse host responses in recipient cells. Although backbone modules of conjugative plasmids of several bacteria species have been documented (Fernández-López et al., 2006; Norman and Hansen, 2009; Pachulec and van der Does, 2010), the backbone modules of the *A. salmonicida* pRAS family of R plasmids have not been characterized. In the absence of such information, mechanisms underlying the conjugative transfer of *A. salmonicida* plasmids remain unknown. Also, it is unknown whether the *A. salmonicida* conjugative plasmids share similar backbone modules with plasmids of other bacteria species.

In Norway, *A. salmonicida* isolates collected before the rigorous vaccination program was introduced in the mid-1990s, that significantly reduced furunculosis cases and antibiotic use, carried a diverse repertoire of pRAS plasmids (Markestad and Grave, 1997; Reith et al., 2008). L’Abée-Lund and Sørum (2002) observed that the three largest plasmids pRAS1, pRAS2, and pRAS4 whose size varied from 45 to 50 kbp were all self-transmissible unlike the small mobilizable but non-self-transmissible pRAS3. Among the large self-transferable conjugative plasmids of the furunculosis agent *A. salmonicida* subsp. *salmonicida*, pRAS4 is the least studied, with limited sequence data available, and no complete published annotation (L'Abée-Lund and Sørum, 2000, 2001; L’Abée-Lund and Sørum, 2002; Sørum et al., 2003). Thus, in this study, we sought to characterize the composition of genes that form the backbone modules of pRAS4 using two plasmids found in the genomes of archived *A. salmonicida* strains F878/91 and F830/91 isolated from Atlantic salmon (*Salmo salar* L) showing characteristic signs of furunculosis in 1991. In addition, we wanted to determine whether the backbone modules of the two pRAS4 plasmids from *A. salmonicida* strains F878/91 and F830/91 were comparable with the backbone modules of conjugative plasmids found in other bacteria species. We envisage that data generated in this study will shed new insight into the type of conjugative machinery used by pRAS4 in self-transfer and spreading of AMR genes. We identify the conjugation machinery genes that had previously eluded annotations, demonstrate self-transfer of pRAS4 by mating, and show that the pRAS4 replicon does not belong to the IncU group to which the related plasmid pRAS1 was assigned, but instead belongs to a previously uncharacterized plasmid backbone of environmental origin.

## 2 Materials and methods

### 2.1 Bacterial strains and culture

Two archived *A. salmonicida* strains F878/91 and F830/91, were obtained from the −80 °C freezer. Both archived strains were collected from diseased farmed Atlantic salmon showing characteristic clinical signs of furunculosis at the Western Coast of Norway in 1991. They were cultured on Blood Agar (BA) followed by culture in Tryptic Soy Broth (TSB) at 14 °C under aerobic conditions. A plasmid agarose gel prepared in 1991 is included in this study to show the pRAS4 plasmid. The *Aeromonas salmonicida* strain 1993/91 was used as donor of pRAS4 to *Escherichia coli* DH5α.

### 2.2 Antimicrobial susceptibility testing and minimum inhibition concentration determination

Antimicrobial susceptibility testing (AST) was performed for both *A. salmonicida* strains F878/91 and F830/91. The disc diffusion assay was conducted on Mueller-Hinton (MH) agar plates following the standard procedure described by Kirby-Bauer (Hudzicki, 2009) and the National Committee for Clinical Laboratory Standards (Watts et al., 2008). The discs used were tetracycline (30 μg), trimethoprim (5 μg), and compound sulphonamides (300 μg, total containing sulphamerazine 78.6 μg, sulphathiazole 109.42 μg and sulphadiazine 110.53 μg) (Oxoid, UK). The susceptibility breakpoints for all antibiotics were recommended by CLSI (Watts et al., 2008), whereas no standardized CLSI breakpoints are available for *Aeromonas salmonicida*. Therefore, interpretation of susceptibility for *A. salmonicida* was performed with reference to published guidelines for aquatic animal pathogens (Smith, 2006). The same protocol for the antibiotic susceptibility testing by disc diffusion was used for the *E. coli* transconjugants following conjugation experiments.

The Minimum Inhibitory Concentrations (MICs) were determined using broth microdilution for the same antibiotics (tetracycline, sulphonamides, and trimethoprim) described above and were determined using the microdilution method in sensititre custom plates (Thermo Fisher Scientific, Waltham, MA, United States). The concentrations of each bacteria suspension were adjusted to 0.5 McFarland equivalent using the nephelometer and the microtiter trays with *A. salmonicida* incubated at 15 °C for 72 h. Each MIC was determined as the lowest concentration of antibiotic agent inhibiting visible bacterial growth and expressed in μg/mL.

### 2.3 Conjugation experiments

Conjugation experiments were carried out using a plate mating approach as previously described (Sørum et al., 2003). Donor *Aeromonas salmonicida* donors (F878/91, F830/91 and 1993/91), revived directly from −80 °C and the recipient *Escherichia coli* DH5α were grown overnight on non-selective blood agar at 14 °C for the donors and at 37 °C for the recipient. A loopful of donor and recipient cultures was mixed in approximately equal proportions, and spotted onto non-selective blood agar plates, followed by incubation at room temperature (~22 °C) for 24 h to facilitate cell-to-cell contact and plasmid transfer.

After mating, the bacterial growth from the mating area was collected and resuspended in sterile saline. The suspension was adjusted to a 0.5 McFarland standard to standardize cell density and subsequently spread onto Mueller-Hinton agar plates supplemented with tetracycline (30 μg/mL). Plates were incubated at 37 °C for 24 h. Under these conditions, *E. coli* DH5α grows readily while *Aeromonas salmonicida* which is psychrotrophic and grows optimally at 14 °C, is strongly disfavored. Colonies displaying *E. coli*-like morphology were picked and re-streaked onto fresh MH agar containing tetracycline (30 μg/mL) at 37 °C to obtain single-colony-purified transconjugants.

To confirm that the antimicrobial resistance phenotype had been transferred, antimicrobial susceptibility testing (AST) was performed on the purified transconjugants using the disc diffusion protocol described in section 2.2, with tetracycline (30 μg), sulphonamides (300 μg) and trimethoprim (5 μg) discs on MH agar incubated at 37 °C for 24 h. Transfer frequencies of pRAS4 were determined for the donors *A. salmonicida* subsp. *salmonicida* F878/91 and 1993/91 in four independent mating trials performed between 18th August and 22nd September 1993, with each trial conducted in parallel on agar surface and in broth. For each trial, viable donor and recipient counts were determined by serial dilution and plate counting on non-selective blood agar (donors at 15 °C, recipients at 37 °C). Transconjugants were enumerated by counting tetracycline-resistant *E. coli*-like colonies recovered on Mueller-Hinton agar containing tetracycline (30 μg/mL) at 37 °C. Transfer frequencies were expressed as the number of transconjugants per recipient cell. Where no transconjugants were recovered the value is reported as “nd” (not detected).

To visualize pRAS4 both in the donor *A. salmonicida* subsp. *salmonicida* 1993/91 and in a transconjugant *E. coli* DH5α from the mating on solid surface plasmid purification and agarose electrophoresis was performed as described in Sørum et al., 1993, 2000, 2003. Two *E. coli* DH5α transconjugants from a parallel mating experiment with *Aeromonas salmonicida* subsp. *salmonicida* 1995/91 with pRAS1 was included in the same gel. In addition, plasmids from two *Aeromonas salmonicida* subsp. *salmonicida* isolates (33DK and 2442/89) without R plasmids were included in the agarose gel.

### 2.4 DNA extraction

Genomic DNA (gDNA) was extracted from culture grown in Tryptic Soy Broth (TSB) at 14 °C for 72 h, for the two donors *A. salmonicida* (F878/91 and F830/91). Both donors were cultured in TSB to generate a concentration of 10^9^ CFU/mL. Cells were harvested by centrifugation at 5,000 × *g* for 10 min at 4 °C. The gDNA was extracted using MagAttract® High Molecular Weight (HMW) DNA extraction Kit according to the manufacturer’s instructions (Qiagen, Germany). Briefly, each bacterium was harvested after centrifugation in 2 mL Eppendorf tubes, and the pellets were resuspended in 180 ul ATF buffer. To each tube 20 μL proteinase K was added followed by incubation at 56 °C for 30 min. This was followed by adding 4 μL RNAse, 15 μL of Mag Attract suspension G and 289 ul Buffer MB to each vial. The suspension from each tube was transferred onto a MagAttract holder containing gDNA and were separated after incubation for 60 s which separated the supernatant without disturbing the beads at room temperature. DNA was purified using a silica column-based method. After binding, the column was washed sequentially with Wash Buffer (MW1) followed by ethanol-based Wash Buffer PE to remove residual contaminants. The column was then dried by centrifugation prior to DNA elusion. Thereafter, gDNA was harvested by elution in 100 μL elution buffer (EB). The gDNA integrity was determined using 1% agarose gel electrophoresis and NanoDrop spectrophotometer (Thermo Fisher Scientific, Waltham, MA, United States). The gDNA concentration was measured using the Qubit fluorometer (Thermo Fisher Scientific, Waltham, MA, United States) using the dsDNA High Sensitivity (HS) assay based on the manufacturer’s guidelines (Life Technologies Inc., Carlsbad, CA, United States).

### 2.5 Whole genome sequencing

High-quality gDNA extracted from *A. salmonicida* strains F878/91 and 830/91 was used for whole genome sequencing (WGS). Sequencing libraries were prepared using an Illumina-compatible protocol (e.g., Nextera or equivalent) and run as paired end reads (2 × 150 bp) on an Illumina platform. Libraries were prepared using the Nextera DNA flex Tagmentation using the paired end genome libraries (Illumina Inc. San Diego, CA, USA)(Gaio et al., 2019). The Qubit® DNA HS Assay Kit in a Qubit fluorometer (Thermo Fisher Scientific, Waltham, MA, United States) was used to quantify the Illumina libraries while Agilent 2,100 Bioanalyzer System using the Agilent HS DNA Kit (Agilent Technologies, CA, United States) was used to determine the size of the library fragments. The Illumina sequencing was done using the V3 reagent kits using paired end read lengths of 2 × 300 bp using Illumina MiSeq (Illumina Inc., USA) (Kaspersen et al., 2020). Raw reads were inspected using FastQC to assess per-base quality scores, adapter contamination, GC content, and overrepresented sequences using the online Galaxy platform^1^ version 21.05. Low quality bases and residual adapters were removed using Trimmomatic using the FastQC Version 0.11.9 software (Bolger et al., 2014; Andrews, 2017). The filtered reads were assembled using the Unicycler on the Galaxy Australia platform.^2^ Genome annotation was done using the prokaryotic genome annotation pipeline (PGAP) (Tatusova et al., 2016) from the National Center for Biotechnology and Infection (NCBI) and Prokka (Seemann, 2014).

### 2.6 Bioinformatic identification of plasmids and antimicrobial resistance genes

The complete genome sequences of *A. salmonicida* strains F878/91 and F830/91 together with their plasmid sequences were annotated using the independent pipelines, Prokka (v1.14.x) and Bakta (v1.8). The identification of plasmids was done using Plasmidfinder v 2.0 set at a threshold of 80% (Ullah et al., 2020) while the Proksee software was used to generate the plasmid circular and linear maps (Seemann, 2014). The identification of AMR genes was done using Staramr version 0.7.2 and ABRicate version 1.0.1 in the Comprehensive Antimicrobial Resistance Database (CARD) and ResFinder software set at a threshold of 80% (Seemann, 2014; Alcock et al., 2020; Tran et al., 2021).

### 2.7 Bioinformatic re-annotation of pRAS4 conjugation modules

To resolve the assignments of conjugation-related coding sequences (CDSs) in pRAS4 beyond the initial Prokka annotation, candidate protein sequences were subjected to domain-based and similarity-based re-analysis. Protein sequences were retrieved from the Prokka-annotated GenBank files of pRAS4-F878/91 and pRAS4-F830/91 and submitted to InterProScan version 5.77–108.0 via the European Bioinformatics Institute (EBI) web server,^3^ with all member database enabled including Pfam, CDD, PANTHER, SUPERFAMILY, Gene3D, TIGRFAMS, HAMAP, ProSite Profiles and Patterns, SMART, Phobius, SignalP and TMHMM. Domain assignments, InterPro signatures, and predicted transmembrane topology were recorded for each protein, together with E-values reported by each member database.

Sequence similarity searches were performed using BLAST against the NCBI non-redundant protein sequences (nr) database (clustered nr) at default settings.^4^ Top hits were recorded with their accession numbers, percent identities, query coverage, and E-values. Proteins of interest included the 823-amino acid ORF originally annotated by Prokka as TraN (located at complement (13,997..16,468) in pRAS4-F878/91 and at 14,467..16,938 in pRAS4-F830/91), and the 366-amino-acid hypothetical protein common on both plasmids (located at 7,375..8,475 in pRAS4-F878/91 and at complement (22,460..23,560) in pRAS4-F830/91).

## 3 Results

### 3.1 Antimicrobial susceptibility

Both *A. salmonicida* donor strains tested using the disc diffusion method, were resistant to tetracycline, but susceptible to compound sulphonamides and trimethoprim (Table 1). Compound sulphonamides and trimethoprim were included in the susceptibility panel to assess the potential presence of compound sulphonamides and trimethoprim resistance determinants commonly associated with multidrug resistance plasmids in *A. salmonicida,* although neither of the donor strains exhibited phenotypic resistance. The recipient *E. coli* DH5α was susceptible to all four antibiotics tested. The transconjugants derived from strain F878/91-DH5α and F830/91-DH5α exhibited resistance to tetracycline but remained susceptible to compound sulphonamides, and trimethoprim, indicating selective acquisition of tetracycline resistance. To further validate the specificity of plasmid transfer, colonies recovered from tetracycline and compound sulphonamides inhibition zones were screened by antimicrobial susceptibility testing. The recovered transconjugant consistently exhibited tetracycline resistance while remaining susceptible to compound sulphonamides, and trimethoprim, confirming transfer of pRAS4 encoded tetracycline resistance. These results indicate successful transfer of tetracycline resistance from donors F878/91 and F830/91, consistent with transfer of pRAS4.

**Table 1:** Antimicrobial susceptibility tests for the donors, recipient, and transconjugant strains.

Strain	Tetracycline	Compound sulfonamides	Trimethoprim
*Aeromonas salmonicida* F878/91 (donor)	R	S	S
*Aeromonas salmonicida* F830/91 (donor)	R	S	S
*Aeromonas salmonicida* 1993/91 (donor)	R	S	S
*Escherichia coli* DH5α (recipient)	S	S	S
F878/91-DH5α (Transconjugant)	R	S	S
F830/91-DH5α (Transconjugant)	R	S	S

### 3.2 Minimum inhibition concentration

Table 2 shows the MICs for tetracycline, sulfamethoxazole, and trimethoprim determined for both donor strains, the recipient *E. coli* DH5α, and both the transconjugants. Among the donors, strain F878/91 and F830/91 showed MICs of 16 μg/mL for tetracycline, ≤0.25 μg/mL for sulphamethoxazole and trimethoprim. The recipient *E. coli* DH5α showed MICs at or below the lowest concentrations tested for all three antibiotics (≤0.25 μg/mL sulphamethoxazole, ≤0.25 μg/mL trimethoprim, and ≤0.25 μg/mL tetracycline). Both transconjugants F878/91-DH5α and F830/91-DH5α exhibited tetracycline MIC (16 μg/mL) identical to their donor strains but remained susceptible to sulfamethoxazole and trimethoprim (≤0.25 μg/mL), indicating selective transfer of tetracycline resistance determinants. These MIC results are consistent with the plasmid content identified by phenotypic properties of AST where only tetracycline resistance as single resistance phenotype is associated with pRAS4 plasmid.

**Table 2:** Minimum inhibitory concentrations for the donors, recipient, and transconjugant strains.

Strain	Tetracycline (μg/mL)	Sulfamethoxazole (μg/mL)	Trimethoprim (μg/mL)
*Aeromonas salmonicida* F878/91	16	≤0.25	≤0.25
*Aeromonas salmonicida* F830/91	16	≤0.25	≤0.25
*Escherichia coli* DH5α	≤0.25	≤0.25	≤0.25
F878/91-DH5α	16	≤0.25	≤0.25
F830/91-DH5α	16	≤0.25	≤0.25

≤ MIC values represent the lowest concentration inhibiting visible growth. Values reported as ≤ indicate inhibition at the lowest concentration tested.

### 3.3 Conjugation experiments

Mating experiments between donors *A. salmonicida* strains F878/91, F830/91 and 1993/91 and the recipient *E. coli* DH5α produced antibiotic-resistant transconjugants on Mueller–Hinton (MH) agar supplemented with tetracycline (30 μg). Successful conjugation required the use of fresh donor cells revived directly from −80 °C glycerol stocks; earlier mating attempts using donor working stocks maintained at 4–5 °C had consistently failed to produce transconjugants, whereas freshly revived donor cultures from −80 °C stocks produced successful transfer. *A. salmonicida* is a psychrotrophic organism and prolonged storage of working stocks at refrigeration temperatures has been reported to affect plasmid stability and the expression of conjugation-related genes in related Gram-negative systems (Headd and Bradford, 2020).

**Figure 1:**
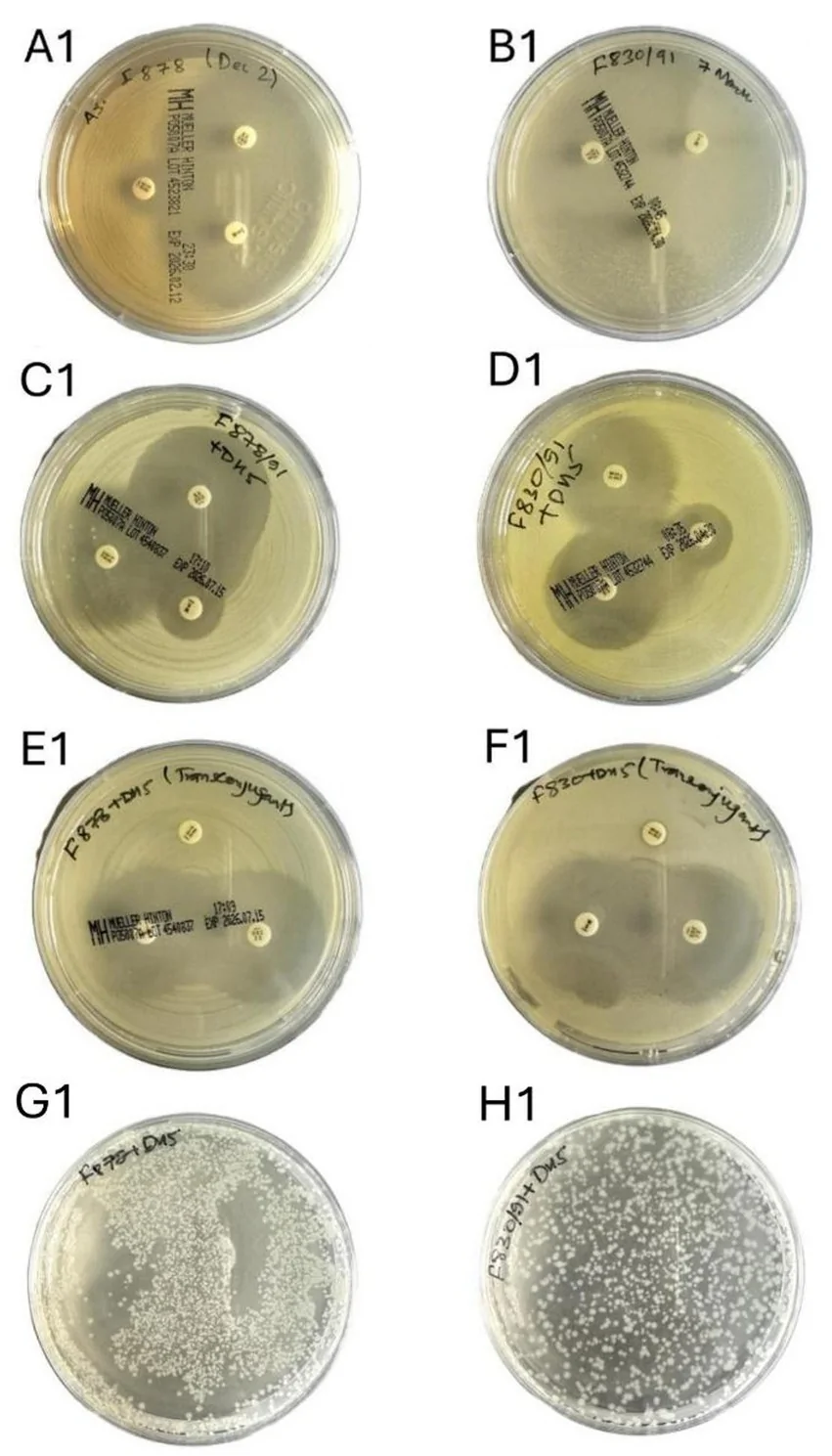
Antimicrobial susceptibility testing of the *Aeromonas salmonicida* subsp. *salmonicida* donors, the *Escherichia coli* DH5α recipient and the resulting transconjugants, and recovery of pRAS4 transconjugants on selective medium. The donor strains (F878/91, F830/91) and the transconjugants are resistant to tetracycline but remain susceptible to compound sulphonamides and trimethoprim, whereas the *E. coli* DH5α recipient is susceptible to all antibiotics tested. The selection plates (Mueller-Hinton agar supplemented with tetracycline, 30 µg/mL) show dense growth of tetracycline-resistant, *E. coli*-like transconjugant colonies recovered after mating, confirming selective transfer of the tetracycline-resistance phenotype encoded by pRAS4.

Figure 1 shows representative results of the mating experiments. Discrete colonies displaying *E. coli-*like morphology were observed on the selection plates for both donor strains. Single colonies from the selection plates were re-streaked onto fresh MH agar containing tetracycline (30 μg) at 37 °C to obtain single-colony purified transconjugants. The transconjugants retained typical *E. coli*-like morphology on the second-pass selection, indicating that they were viable on tetracycline-containing medium under conditions non-permissive for the donor.

The transconjugants derived from both donors *A. salmonicida* F878/91 and F830/91 were resistant to tetracycline and susceptible to sulphonamides and trimethoprim (Figure 1), reproducing the resistance profile observed in the *A. salmonicida* donors (Section 3.1) for tetracycline while leaving the *E. coli* DH5α susceptibility profile to sulphonamides and trimethoprim unchanged. This selective transfer of tetracycline resistance is consistent with the genetic content of pRAS4, which encodes the TetA(A) efflux pump together with its TetR(A) repressor but does not encode sulphonamides or trimethoprim resistance determinants.

**Figure 2:**
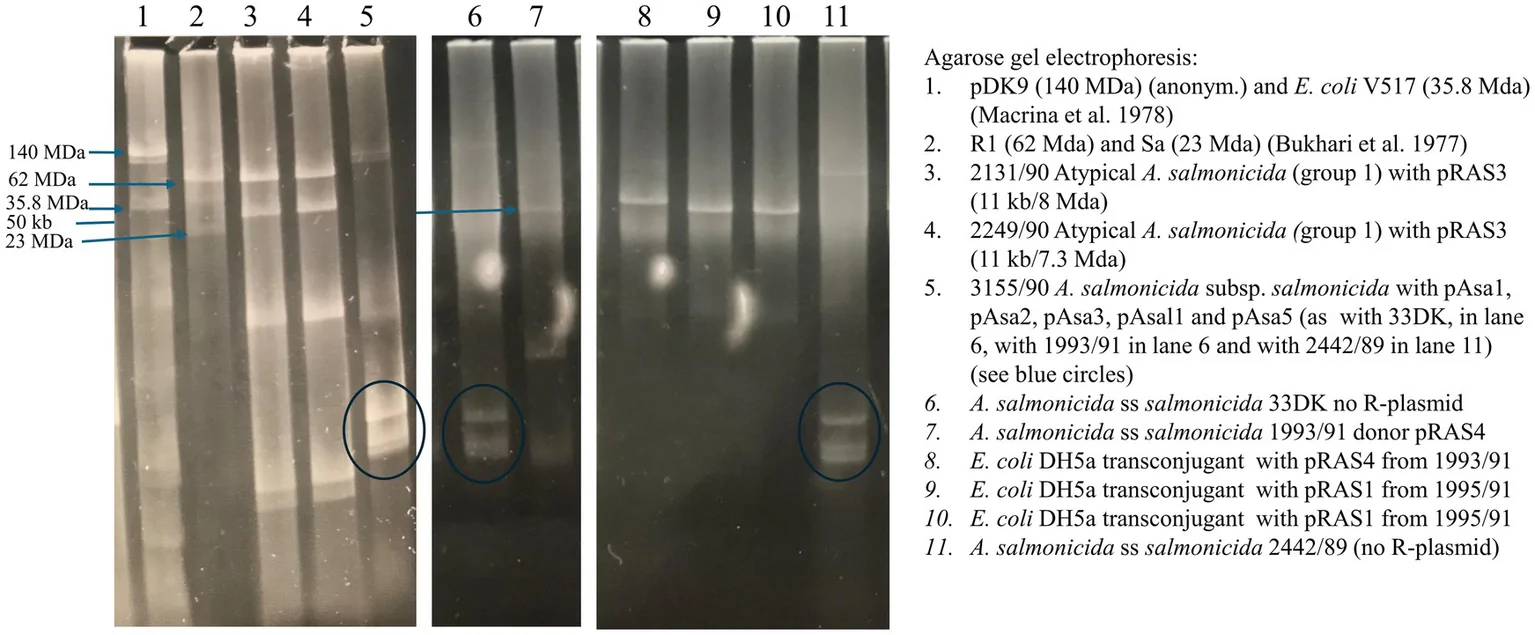
Agarose gel electrophoresis of plasmid DNA demonstrating transfer of a ~50 kb plasmid (pRAS4) from *Aeromonas salmonicida* subsp. *salmonicida* donor 1993/91 to *Escherichia coli* DH5α. Molecular size markers are indicated on the left (140 MDa, 62 MDa, 35.8 MDa, 50 kb, 23 MDa). Lanes: (1) pDK9 (140 MDa) and *E. coli* V517 (35.8 MDa) (Macrina et al., 1978); (2) R1 (62 MDa) and Sa (23 MDa) (Bukhari et al., 1977); (3) *A. salmonicida* atypical strain 2131/90 (group 1) carrying pRAS3 (11 kb/8 MDa); (4) *A. salmonicida* atypical strain 2249/90 (group 1) carrying pRAS3 (11 kb/7.3 MDa); (5) *A. salmonicida* subsp. *salmonicida* 3155/90 carrying pAsal1 (4.2 MDa); (6) *A. salmonicida* subsp. *salmonicida* 33DK, no R-plasmid; (7) *A. salmonicida* subsp. *salmonicida* 1993/91 donor carrying pRAS4; (8) *E. coli* DH5α transconjugant carrying pRAS4 from donor 1993/91; (9, 10) *E. coli* DH5α transconjugants carrying pRAS1 from donor 1995/91; (11) *A. salmonicida* subsp. *salmonicida* 2442/89, no R-plasmid.

The successful production of transconjugants demonstrates that pRAS4 is a self-transmissible conjugative plasmid capable of transferring tetracycline resistance from *A. salmonicida* to *E. coli.* Transfer frequencies of pRAS4 from the donors *A. salmonicida* subsp. *salmonicida* F878/91 and 1993/91, with each trial conducted in parallel on agar surface and in broth (Table 3). Transconjugants were recovered from both donors and on both mating substrates in all trials in which a measurement was made, confirming the self-transmissibility of pRAS4. For the F878/91 donor, transfer frequencies on agar ranged from 2.88 × 10^−2^ to 5.9 × 10^−5^ transconjugants per recipient cell across three trials, and in broth from 2.3 × 10^−5^ to 5.1 × 10^−7^. For the 1993/91 donor, agar transfer frequencies ranged from 6.25 × 10^−2^ to 4.6 × 10^−7^ across four trials, and broth frequencies ranged from 8.85 × 10^−6^ to 7.5 × 10^−6^. For both donors, transfer frequencies on the agar surface were consistently one to four orders of magnitude higher than the corresponding broth frequencies, indicating that conjugative transfer of pRAS4 is strongly favored by surface mediated mating, as is characteristic of plasmids encoding MPF_T-type (VirB/VirD4) machinery. Agarose gel electrophoresis demonstrated that *A. salmonicida* subsp. *salmonicida* donor 1993/91 was able to transfer a 50 kb plasmid to *E. coli* DH5α (Figure 2).

**Table 3:** Transfer frequency of pRAS4 from *Aeromonas salmonicida* subsp. *salmonicida* donors F878/91 and 1993/91 to *Escherichia coli* DH5α in four independent mating trials performed in 1993.

Donor strain	Trial 1 Agar	Trial 1 Broth	Trial 2 Agar	Trial 2 Broth	Trial 3 Agar	Trial 3 Broth	Trial 4 Agar	Trial 4 Broth
F878/91	2.88 × 10^−2^	2.3 × 10^−5^	3.2 × 10^−4^	1.6 × 10^−5^	5.9 × 10^−5^	5.1 × 10^−7^	nd	nd
1993/91	6.25 × 10^−2^	nd	4.6 × 10^−7^	8.85 × 10^−6^	6.4 × 10^−5^	7.5 × 10^−6^	6.46 × 10^−5^	nd

Values are transconjugants per recipient cell; nd, not detected.

### 3.4 Whole genome sequencing of *Aeromonas salmonicida* strains F878/91 and F830/91

**Figure 3:**
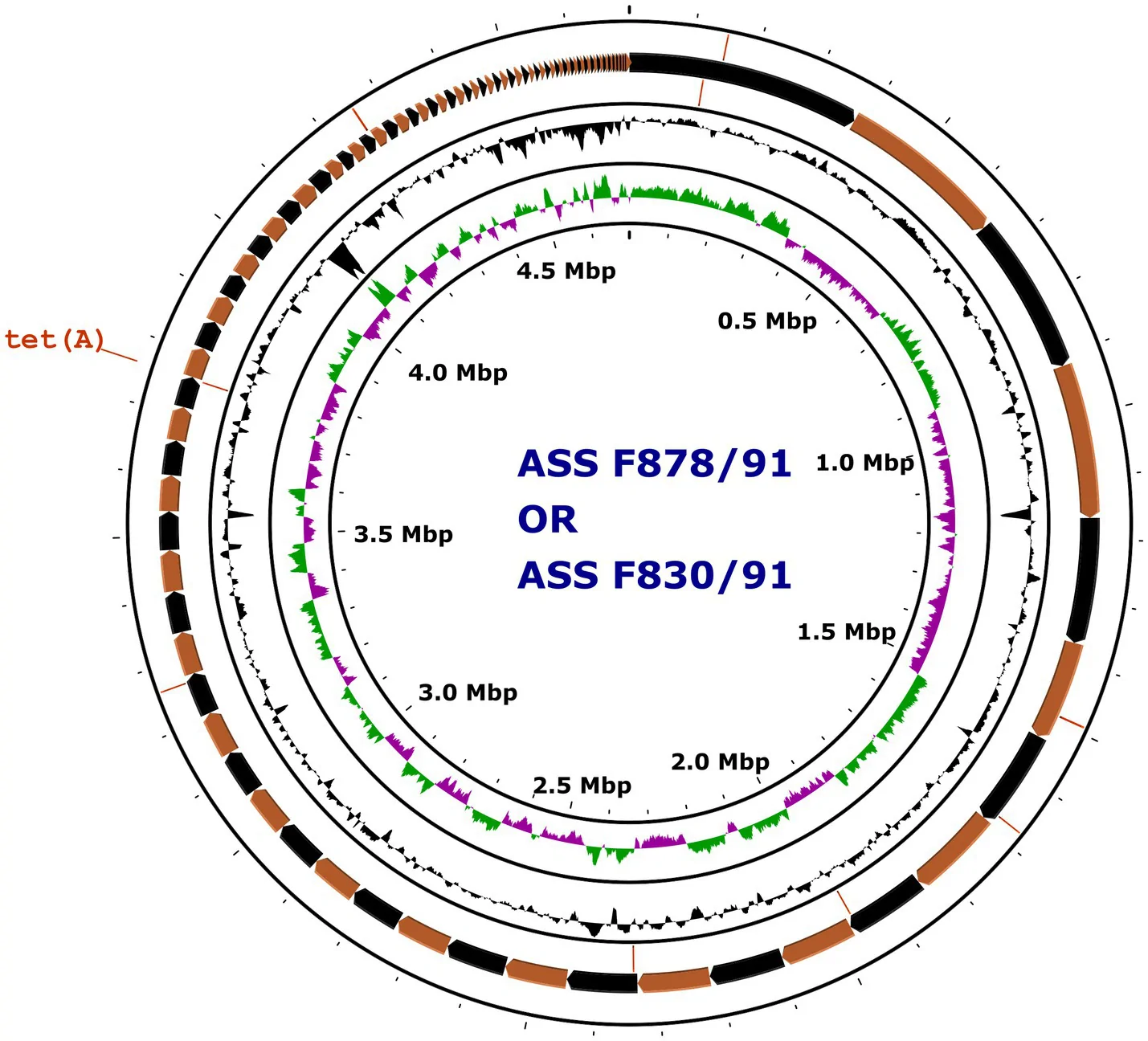
Circular whole-genome map of *Aeromonas salmonicida* subsp. *salmonicida* strains F878/91 and F830/91 generated in Proksee. From the outer to the inner rings the map shows annotated coding sequences on the forward and reverse strands, genome position in megabase pairs (Mbp), GC content and GC skew. The location of the *tet*(A) tetracycline-resistance determinant is indicated.

Table 4 shows WGS results of *A. salmonicida* strains F878/91 and F830/91 that had genome sizes of 4.6 and 4.7 Mbp, respectively. The guanosine or cytosine (GC) content, scaffolds, protein and gene contents for both strains are shown in Table 4. Both *A. salmonicida* strains F878/91 and F830/91 had a high proportion of uncharacterized hypothetical genes accounting for 34.24% (1,634/4509) and 36.33% (1,659/4567), respectively. The circular maps of *A. salmonicida* strains F878/91 and F830/91 are shown in Figure 3.

**Table 4:** Whole genome sequence data of *Aeromonas salmonicida* strains F878/91 and F830/91.

Parameters	*Aeromonas salmonicida* F878/91	*Aeromonas salmonicida* F830/91
Size Mb	4.6	4.7
GC content	58.5	58.5
Scaffold	96	160
CDS	4,409	4,468
Genes	4,509	4,567
Hypothetical genes	1,634	1,659
Proportion of hypothetical genes	34.24%	36.33%
Plasmids	pRAS4 + pAsa1 + pAsa2 + pAsa3 + pAsal1 + pAsa5	pRAS4 + pAsa1 + pAsa2 + pAsa3 + pAsal1 + pAsa5
NCBI Accession number	PX997076	PZ097580

### 3.5 Characterization of pRAS4 of *Aeromonas salmonicida* strains F878/91 and F830/91

**Figure 4:**
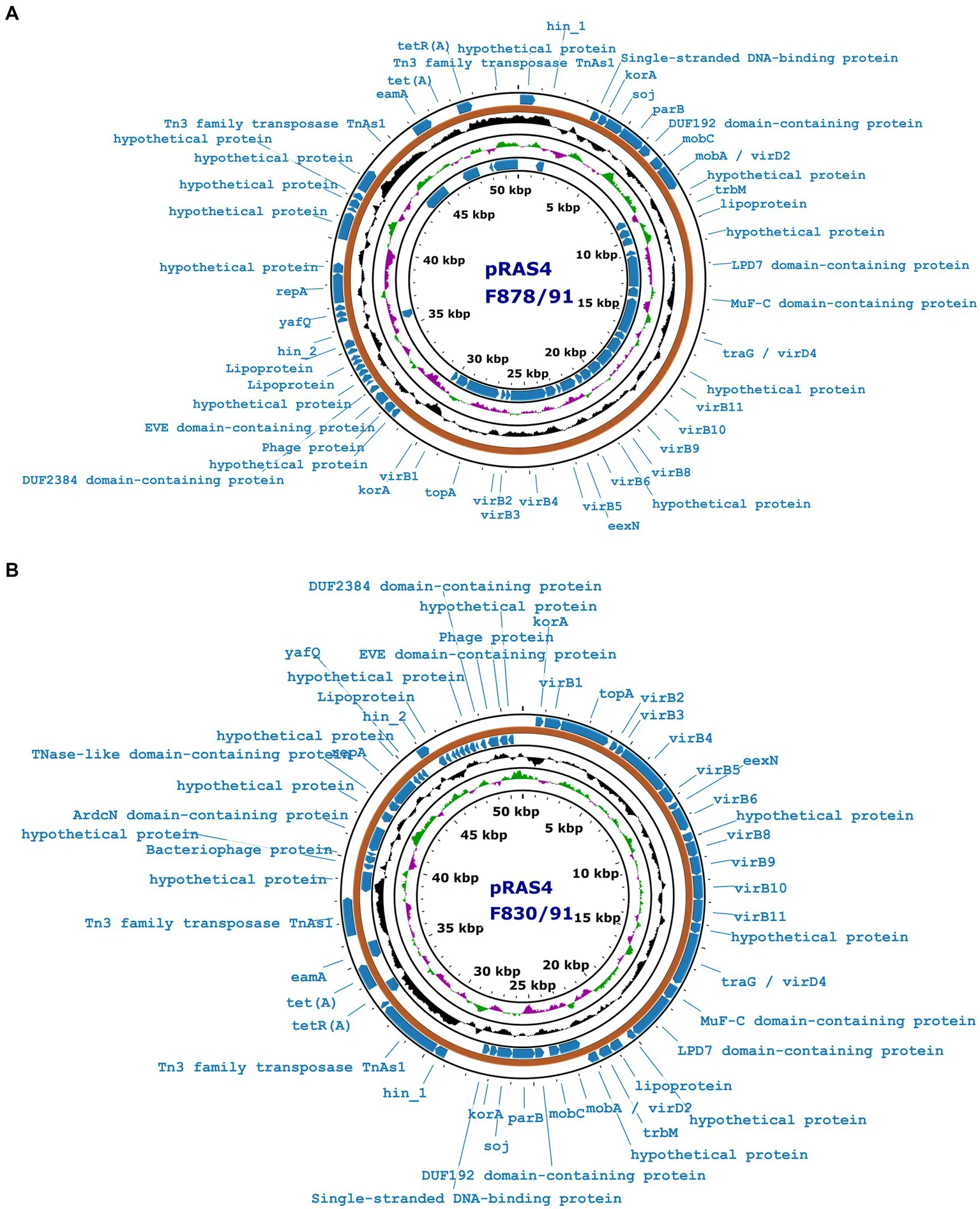
Circular maps of the pRAS4 plasmid from *Aeromonas salmonicida* subsp. *salmonicida* strains F878/91 **(A)** and F830/91 **(B)**, generated in Proksee. Concentric rings display the annotated coding sequences, including the *virB1–virB11* mating pair formation cluster, the *traG/virD4* type IV coupling protein, the *mobA/virD2* relaxase and *mobC* relaxosome gene, replication (*repA*) and partitioning (*parA/parB, soj*) genes, maintenance and regulatory genes, and the *tet(A)/tetR(A)* tetracycline-resistance cassette with its flanking Tn*3*-family transposase (*TnAs1*) copies. Plasmid position is marked in kilobase pairs (kbp).

**Figure 5:**
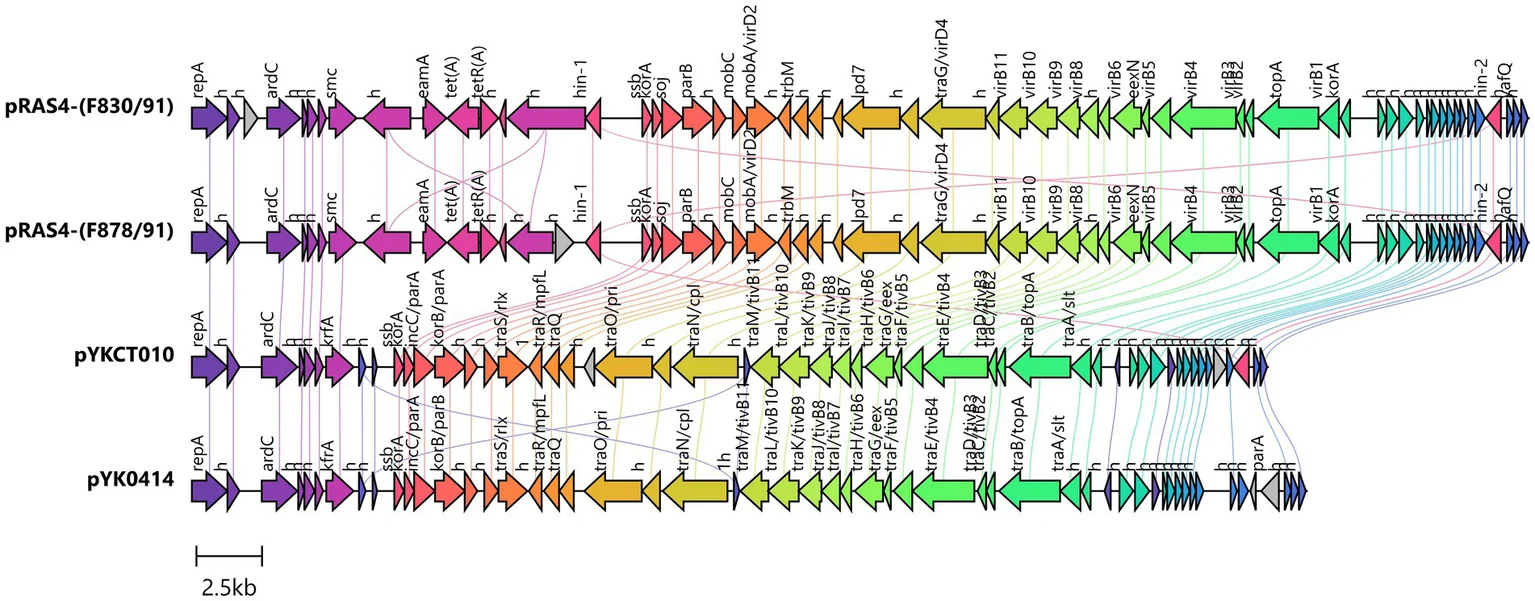
Linear comparison of gene content and synteny among four plasmids: pRAS4-F830/91 and pRAS4-F878/91 from *Aeromonas salmonicida* subsp. *salmonicida* (Norway, 1991) and pYKCT010 and pYK0414 from uncultured bacteria of Japanese river sediments (2021). Coloured arrows represent predicted genes and proteins, with connecting bands indicating regions of homology and conserved gene synteny across the four plasmids. All four plasmids share a conserved backbone comprising the *virB1-virB11* T4SS cluster (lacking *virB7*), the type IV coupling protein, relaxase, *mobC*, replication (*repA*) and partitioning genes. The two pRAS4 plasmids additionally carry an operon encoding two topoisomerase A (*topA*) genes, the EamA efflux transporter and the TetA efflux pump with its TetR(A) repressor, flanked by Tn3-family transposases, that is absent from pYKCT010 and pYK0414. Scale bar, 2.5 kb.

The complete sequences of the pRAS4-F878/91 and pRAS4-F830/91 recovered from *A. salmonicida* strains F878/91 and F830/91 were both estimated to be 50 kbp (Table 4). Annotation using Prokka (v1.14.x) and Bakta (v1.8) identified 57 predicted coding DNA sequences (CDS) distributed across the forward and reverse strands, 57 genes, and 32 hypothetical genes for both pRAS4-F878/91 and pRAS4-F830/91. Figures 4A,B show the circular map of pRAS4-F878/91 and pRAS4-F830/91 from *A. salmonicida* strains F878/91 and F830/91, respectively. A comparison of the composition and organization of genes in pRAS4-F878/91 and pRAS4-F830/91 that includes genes that form the type IV secretion system (T4SS), mating pair formation (MPF), antitoxin, and AMR genes is shown in Figure 5. Table 4 also shows the size, GC content, CDS, total genes and hypothetical genes encoded by all plasmids of *A. salmonicida* strains F878/91 and F830/91.

As shown in Figure 5, both pRAS4-F878/91 and pRAS4-F830/91 encoded an operon of 10 virB proteins that form the canonical T4SS nanomachinery consisting of virB1, virB2, virB3, virB4, virB5, virB6, virB8, virB9, virB10, and virB11. At the level of the original Prokka annotation, neither a canonical *virB7* nor a *virD4* was assigned within the virB operon; the resolution of this provisional finding by domain-based re-annotation is presented in Section 3.6. Figure 5 shows the hypothetical T4SS nanomachinery of pRAS4 based on the identified virB proteins in pRAS4-F878/91 and pRAS4-F830/91. Other genes found in the virB operon included transfer (*traI*), the entry exclusion (*eexN*), and DNA topoisomerase I (*topA*). Note that *eexN* was placed between *virB5* and *virB6* while *traI* was placed between *virB6* and *virB8*. As shown in Figure 5, upstream of the virB operon were genes associated with the MPF that included transfer (*tra*) genes like *traQ*, *traN*, *traS*, *traO* and *trbM*. Among the plasmid mobilization (*MOB*) genes only *mobC* was found in pRAS4-F878/91 and pRAS4-F830/91.

Other genes found upstream of the virB operon in both pRAS4-F878/91 and pRAS4-F830/91 include the single stranded binding (*ssB*) protein, transfer DNA invertase 1 (*hin I*), DNA invertase II (*hin II*), kil-override A (*korA*), partitioning A (*parA*), partitioning B (*parB*), replication initiator protein (*repA*), antirestriction protein type C (*ardC*), suppression of J (*soj*) and *YafQ* toxin (Figures 4A,B). In addition, both pRAS4-F878/91 and pRAS4-F830/91 encoded the EamA efflux transporters and the TetA efflux pump together with the tetracycline repressor TetR, topoisomerase A (*topA*), the *smc* gene and several hypothetical (*h*) genes (Figure 5) (see Table 5).

**Table 5:** Characterization of plasmids found in *Aeromonas salmonicida* strains F878/91 and F830/91.

Strain	Plasmids	Size	GC content (%)	Scaffold	CDS	Genes	Hypothetical genes
*Aeromonas salmonicida* F878/91	*pRAS4-F878/91*	~50 kb	57	1	57	57	32
*Aeromonas salmonicida* F878/91	*pASal1-F830/91*	~6.5 kb	54.6	2	6	6	1
*Aeromonas salmonicida* F878/91	*pAsa1-F878/91*	~5.4 kb	57	1	10	7	3
*Aeromonas salmonicida* F878/91	*pAsa2-F878/91*	~5.2 kb	52	1	5	4	1
*Aeromonas salmonicida* F878/91	*pAsa3-F878/91*	~5.4 kb	54.2	1	8	8	1
*Aeromonas salmonicida* F878/91	*pAsa5-F878/91*	~100.8 kb	52.5	12	132	132	36
*Aeromonas salmonicida* F830/91	*pRAS4-F830/91*	~50 kb	57	1	57	57	32
*Aeromonas salmonicida* F830/91	*pAsal1-F830/91*	~4.9 kb	55	7	8	8	2
*Aeromonas salmonicida* F830/91	*pAsa1-F830/91*	~5.4 kb	57	1	10	7	3
*Aeromonas salmonicida* F830/91	*pAsa2-F830/91*	~5.2 kb	52	1	6	5	1
*Aeromonas salmonicida* F830/91	*pAsa3-F830/91*	~5.4 kb	54.2	1	8	8	1
*Aeromonas salmonicida* F830/91	*pAsa5-F830/9*	~136.9 kb	54	24	165	165	48

pAsal1 and pAsa5 were identified as fragmented contigs in whole genome assemblies, most likely due to limitations of short-read Illumina sequencing in resolving repetitive regions and structurally complex plasmids. Reported sizes for pAsa5 represent cumulative contig lengths and likely underestimate the full plasmid size (~155 kb).

### 3.6 Identification of the type IV coupling protein and the relaxase of pRAS4

To resolve the assignments of conjugation-related coding sequences (CDSs) in pRAS4, two ORFs were subjected to domain-based and similarity-based re-analysis. The first was an 823-amino-acid CDS originally annotated by Prokka as *TraN* (located at complement (13,997..16,468) in pRAS4-F878/91 and at 14,467..16,938) in pRAS4-F830/91. The second was a 366-amino-acid CDS originally as “hypothetical protein” in both strains (located at 7,375..8,475 in pRAS4-F878/91 and at complement (22,460..23,560) in pRAS4-F830/91). Both proteins were 100% identical in amino acid sequence between the two strains.

#### 3.6.1 The 823-amino-acid ORF encodes a TraG/VirD4-family type IV coupling protein

InterProScan analysis of the 823-amino-acid protein returned consistent assignments to the TraG/VirD4 Type IV coupling protein (T4CP) family across six independent member databases. The Pfam signature PF02534 “T4SS-DNA_trsnf” (Type IV secretory system Conjugative DNA transfer), linked to InterPro entry IPR003688 “TraG/VirD4,” matched residues 100–576 with an *E*-value of 5.1 × 10^−99^. The PANTHER family PTHR37937 “Conjugative Transfer: DNA Transport,” linked to InterPro entry IPR051539 “T4SS-coupling proteins (TraD/TraG/VirD4-like),” matched residues 45–593 with an *E*-value of 5.9 × 10^−115^. The CDD profile cd01127 “TrwB_TraG_TraD_VirD4” matched residues 374–482 with an *E*-value of 4.2 × 10^−13^. The protein also returned matches to the P-loop NTPase fold (Gene3D G3DSA:3.40.50.300, InterPro IPR027417, residues 335–570, *E* = 9.1 × 10^−38^; SUPERFAMILY SSF52540, residues 95–572, *E* = 2.4 × 10^−50^), consistent with the AAA+ ATPase architecture characteristic of T4CPs. A canonical Walker A motif (AAPTRSGKG) was present at residues 138–146, within the Pfam PF02534 domain. The predicted membrane topology (TMHMM and Phobius) revealed two N-terminal transmembrane helices at residues 10–32 and 65–87, with a short cytoplasmic N-terminal tail, a short periplasmic loop, and a large cytoplasmic C-terminal domain spanning residues 88–823. This architecture-two N-terminal transmembrane helices anchoring a cytoplasmic AAA+ ATPase domain is the canonical organization of T4CPs of the TrwB/TraG/TraD/VirD4 family (Cabezón et al., 2015; Gomis-Rüth et al., 2001).

BLAST searches against the NCBI non-redundant protein database confirmed this assignment. The top hits returned uniformly high identity matches characterized T4CPs and TraG/VirD4 family proteins. The closest match was to the type IV secretory system conjugative DNA transfer family protein WP_311230474.1 from unclassified *Acidovorax*, with 89.36% amino acid identity over 100% query coverage (*E* = 0.0). The next-closest matches were to homologous T4CPs from *Burkholderiales* (WP_154048556.1, 88.94% identity), *Collimonas fungivorans* (WP_012274943.1, 73.97% identity), *Microvirgula aerodenitrificans* (WP_281661199.1, 68.74% identity), *Methylomonas* spp. (WP_171697896.1, 67.11% identity), and the previously reported type IV coupling protein TraG/VirD4 of an uncultured bacterium (BBE29073.1, 62.44% identity), with all top 100 hits annotated as Type IV secretory system conjugative DNA transfer proteins or as TraG/VirD4 family coupling proteins. Notably, one hit in the top 100 (CAC79168.1) was deposited as “TraN protein” but shares the TraG/VirD4 domain of similar mis-annotations through public sequence databases and providing a likely explanation for the original Prokka assignment of this ORF as TraN. Taken together, the multi-database domain analysis, the conserved AAA+ ATPase architecture with Walker A motif, the bipartite transmembrane topology, and the uniformity TraG/VirD4 family BLAST hits establish that this 823-amino-acid ORF encodes a Type IV coupling protein of the TraG/VirD4 family, and not a TraN mating-pair stabilization protein. The CDS is hereafter referred to as the T4CP of pRAS4.

#### 3.6.2 The 366-amino-acid hypothetical protein encodes a MobA/VirD2 family relaxase

InterProScan analysis of the 366-amino-acid hypothetical protein returned as a single Pfam domain hit, PF03432 “Relaxase,” corresponding to the MobA/VirD2-like nuclease domain and linked to InterPro entry IPR005094 “Endonuclease MobA/VirD2,” at residues 98-212 with an *E*-value of 4.7 × 10^−14^. A gene 3D match (G3DSA:3.30.930.30, residues 85–223, *E* = 1 × 10^−7^), consistent with the relaxase nuclease fold, supported this assignment. The catalytic nuclease domain was flanked by predicted disordered regions in the N and C terminal tails, an organization typical of MOB-family relaxases (Garcillán-Barcia et al., 2009).

BLAST searches confirmed the relaxase identity. The top hits were uniformly annotated as relaxase or mobilization nuclease domain-containing proteins. The closest match was to the relaxase WP_311230468.1 from unclassified *Acidovorax* (88.80% identity, 100% query coverage, *E* = 0.0), followed by the relaxase WP_012274938.1 from *Collimonas fungivorans* (89.07% identity), WP_281661193.1 from *Microvirgula aerodenitrificans* (80.60%), WP_416146369.1 from *Pseudomonas aeruginosa* (76.12%), and WP_426197137.1 from *Massilia* sp. (71.66%). All top 100 BLAST hits were annotated as relaxase or mobilization nuclease domain-containing proteins. The domain assignment, the presence of the canonical MobA/VirD2 nuclease fold, and the uniformly relaxase-family BLAST hits establish that this 366-amino-acid ORF encodes a relaxase of the MobA/VirD2 family. The CDS is hereafter referred as the relaxase of pRAS4.

#### 3.6.3 The conjugation machinery of pRAS4 is complete and its closest homologs derive from a single environmental plasmid lineage

The identification of the T4CP and the relaxase, together with the previously annotated *virB1-virB11* mating pair formation (MPF) cluster and the *mobC* accessory relaxosome gene, established that pRAS4 encodes a complete set of canonical conjugation genes: a relaxase that initiates plasmid mobilization by nicking the origin of transfer, a T4CP that couples the relaxosome to the secretion channel, an MPF cluster that forms the transmembrane conjugation channel, and the *mobC* relaxosome accessory gene. This molecular complement is consistent with the functional demonstration of pRAS4 self-transfer to *E. coli* DH5α reported in Section 3.2.

The BLAST results for the T4CP and the relaxase consistently identified their closest homologs in the same set of environmental bacteria. For both proteins, the top hits were to a single *Acidovorax* sp. (WP_311230474 for the T4CP, 89.36% identity; WP_311230468 for the relaxase, 88.80% identity), followed by *Burkholderiales*, *Collimonas fungivorans*, *Microvirgula aerodenitrificans*, *Methylomonas* sp., and *Massilia* sp. The consistency of this set of bacteria taxa across both proteins, and the high amino acid identities (89% for both), indicates that the conjugation machinery of pRAS4 belongs to a conserved plasmid backbone shared across diverse environmental β and γ proteobacteria. This protein-level evidence is independent of, and complementary to, the nucleotide-level synteny shared between pRAS4 and the Japanese river-sediment plasmids pYKCT010 and pYK0414 (Section 3.9).

### 3.7 Classification of pRAS4 replication, mobilization and conjugation systems of pRAS4

The replication, mobilization and conjugation systems of pRAS4 were assigned using sequence-and domain-based analyses (Table 6). PlasmidFinder v2.0 at the 80% identity threshold returned no replicon match for pRAS4 across any queried database (Enterobacteriales, Inc18, NT_Rep, Rep1, Rep2, Rep3, RepA_N, RepL and Rep_trans). BLASTp analysis of the 453-amino acid RepA protein against the NCBI non-redundant protein database returned the closest match to RepA of *Acidithiobacillus* sp. (WP_287763939, 90.93% identity, 100% query coverage, *E* = 0.0), followed by RepA proteins of unclassified *Acidovorax* (WP_311230487, 79.35% identity), *Burkholderia stabilis* (WP_412473089, 80.82% identity over 89% query coverage), *Microvirgula aerodenitrificans* (WP_281661184, 70.26% identity) and *Massilia* sp. (WP_426197202, 65.65% identity). No close match was returned to the RepA proteins of the canonical IncU plasmids pRAS1, pAr-32 or pRA3 (Kulinska et al., 2008; Sørum et al., 2003). Notably, the closest *Acidovorax* and *Microvirgula* RepA hits derive from the same plasmids that contain the closest homologs of the pRAS4 T4CP and relaxase (Section 3.6), indicating that the replication initiator, the relaxase and the coupling protein of pRAS4 are co-inherited from a conserved environmental plasmid backbone. Together, the absence of a PlasmidFinder match and the divergence of pRAS4 RepA from the canonical IncU plasmid RepAs indicate that pRAS4 carries a novel replicon that cannot be assigned to any currently named IncU group based on sequence-based replicon typing, and that its closest relatives are environmental plasmids of aquatic β- and γ-proteobacteria.

**Table 6:** Classification of pRAS4 replicon, mobilization, and conjugation system based on sequence and domain-based analyses.

Module/system	Component	Assignment	Evidence
Replication	RepA (453 aa)	Novel replicon; not assignable to any currently named Inc. group	PlasmidFinder v2.0 at 80% identity returned no hit across all queried databases. RepA BLASTp top hit: *Acidithiobacillus* sp. RepA (WP_287763939, 90.93% identity, 100% query coverage); other top hits from environmental plasmids of *Acidovorax*, *Microvirgula*, *Massilia* and *Burkholderia* (65–80% identity). No close match to canonical IncU plasmid RepAs (pRAS1, pAr-32, pRA3).
Relaxosome (MOB)	Relaxase (366 aa)	MOB_P (MobA/VirD2 family)	InterProScan: Pfam PF03432, InterPro IPR005094, *E* = 4.7 × 10^−14^ at residues 98–212. BLASTp top hits: 88–89% identity to characterized relaxases of *Acidovorax*, *Collimonas* and *Microvirgula*.
Relaxosome accessory	MobC (181 aa)	MobC-like	Prokka annotation; no InterProScan domain hit at the default threshold.
Mating pair formation (MPF)	virB1, virB2, virB3, virB4, virB5, virB6, virB8, virB9, virB10, virB11	MPF_T (canonical *virB* type)	Prokka annotation. The 140-aa originally annotated as virB7 returned no TrbC/VirB7 (PF03743) or other T4SS component domain in InterProScan and is not considered a canonical virB7.
Type IV coupling protein (T4CP)	TraG/VirD4 family (823 aa)	TraG/ VirD4 T4CP	InterProScan: Pfam PF02534, InterPro IPR003688, *E* = 5.1 × 10^−99^; PANTHER PTHR37937, *E* = 5.9 × 10^−115^; CDD cd01127, *E* = 4.2 × 10^−13^. BLASTp: 89.36% identity to characterized TraG/VirD4 T4CP of *Acidovorax* sp. (WP_311230474). Two N-terminal TM helices (residues 10–32, 65–87) plus cytoplasmic AAA + ATPase with Walker A motif (AAPTRSGKG, residues 138–146).
Partitioning	parB, soj (parA equivalent)	Type I partitioning system	Prokka annotation
Anti-restriction	ArdC (419 aa)	ArdC-family anti-restriction protein	Prokka annotation in F830 (locus GCNCCJAJ_00041, complement(40575.0.41834)); the same protein is present in F878 at 100% identity but is currently annotated as “hypothetical protein.”
Entry exclusion	eexN	EexN family entry exclusion	Prokka annotation
Maintenance/regulation	korA, yafQ, hin_1, hin_2, ssb, smc	Various regulatory and maintenance functions	Prokka annotation
AMR cassette	tetA(A), tetR(A), eamA, two Tn3-family transposase TnAs1 copies	Tetracycline resistance cassette flanked by Tn3-family transposable elements	Prokka annotation

The mobilization system of pRAS4 was assigned to the MOB_P relaxase family based on the MobA/VirD2-family relaxase identified by InterProScan (Section 3.6), following the MOB classification scheme of the Garcillán-Barcia et al. (2009). The mating pair formation system was assigned to the MPF_T type based on the canonical *virB1-virB11* organization (Guglielmini et al., 2014). The original Prokka annotation of a 140-amino-acid CDS as virB7 was not supported by InterProScan analysis, which returned no TrbC/VirB7 (Pfam PF03743) or other T4SS-component domain hit at the default threshold; this ORF is therefore considered a small lipoprotein of uncertain function rather than a canonical virB7. The maintenance and inheritance stability module of pRAS4 comprises the partitioning genes *parB* and *soj* (parA equivalent), the anti-restriction ArdC, the entry exclusion protein EexN, and additional regulatory and maintenance components annotated by Prokka. The ArdC protein is annotated as such by Prokka in pRAS4-F83/91 [locus GCNCCJAJ_00041, complement (40575..41834)], while the orthologous coding sequence in pRAS4-F878/91 is 100% identical at the amino acid level but is annotated by Prokka as “hypothetical protein”; the two are therefore the same protein. Taken together, pRAS4 is a self-transmissible MOB_P/MPF_T conjugative plasmid carrying a novel replicon of environmental origin.

### 3.8 Additional plasmids in *Aeromonas salmonicida* strains F878/91 and F830/91

In addition to pRAS4, both *A. salmonicida* strains F878/91 and F830/91 harbored several other plasmids, including pAsa1, pAsa2, pAsa3, pAsal1, and pAsa5. pAsa1-F878/91 and pAsa1-F830/91 were identical, with a size of 5,424 bp. In both strains, pAsal1 was fragmented across multiple contigs in the whole genome assemblies; pAsal1-F830/91 was represented by several small contigs, whereas pAsal1-F878/91 was assembled into larger contigs. pAsa2-F878/91 and pAsa2-F830/91 were identical, with a size 5,247 bp, as were pAsa3-F830/91 and pAsa3-F878/91, with a size of 5,463 bp. pAsa5 was also distributed across multiple contigs in both strains, with a cumulative size of approximately 136 kb in pAsa5-F830/91 and 100 kb in pAsa5-F878/91.

### 3.9 Comparison of pRAS4 of *Aeromonas salmonicida* strains F878/91 and F830/91 with other plasmids

Table 7 shows plasmids used in comparison with the two pRAS4 plasmids. BLAST analysis showed high similarity of pRAS4-F878/91 and pRAS4-F830/91 from *A. salmonicida* strains F878/91 and F830/91, respectively, isolated from Atlantic salmon in 1991 in Norway with pYKCT010 and pYK0414 isolated from uncultured bacterium (LC623930.1 and LC623906.1) from river sediments in Japan in 2021 (Figure 5). In terms of size, both pYKCT010 and pYK0414 from river sediments in Japan were approximately 41–42 kb being shorter than the two pRAS4-F878/91 and pRAS4-F830/91 that had a size 50 kb (Figure 3). The difference could be attributed to the inclusion of the EamA efflux transporter, and TetA efflux pumps together with its TetR suppressor, and the two *topA* in pRAS4-F878/91 and pRAS4-F830/91 that were not present in pYKCT010 and pYK0414. Despite this, the sequences of the two pRAS4 plasmids (pRAS4-F878/91 and pRAS4-F830/91) from *A. salmonicida* strains shared 91.19 and 91.14% nucleotide identity with the Japanese pYKCT010 and pYK0414 plasmids (Figure 5). The overall homology for module A and B was 100% for the two pRAS4 plasmids (pRAS4-F878/91 and pRAS4-F830/91) from *A. salmonicida* strains and the Japanese pYKCT010 and pYK0414 plasmids.

**Table 7:** Plasmids and bacteria species used in the study.

Species	Strain	Plasmid	Accession	Location/year
*Aeromonas salmonicida* subsp. *salmonicida*	F878/91	pRAS4-F878/91	PX997076	Norway/1991
*Aeromonas salmonicida* subsp. *salmonicida*	F830/91	pRAS4-F830/91	PZ097580	Norway/1991
Uncultured bacterium from river sediments		pYKCT010	LC623930.1	Japan/2021
Uncultured bacterium from river sediments		pYK0414-12	LC623906.1	Japan/2021

Our findings show that both pRAS4-F878/91 and pRAS4-F830/91 had a similar composition and organization of 10 *virB* genes that form the T4SS nanomachinery with pYKCT010 and pYK0414. As shown in Figure 5, all four plasmids encoded 10 *virB* proteins associated with formation of a T4SS nanomachinery that included virB1, virB2, virB3, virB4, virB5, virB6, virB8, virB9, virB10, and virB11 (Figure 5). All four-plasmids lacked *virB7* within the virB operon. They all had *eexN* between *virB5* and *virB6* and *traI* between *virB6* and *virB8*. In addition, they all had a similar composition and organization of other MPF and coupling genes upstream of the *virB* operon that included the T4CP (re-annotated in Section 3.6 from its original Prokka annotation as *traN*), *traO*, *traS*, and *trbM*. Further, all four plasmids had genes associated with replication (*repA* and *mobC*), partitioning (*parA* and *parB*), and protection against host responses in transconjugants (*ssb*, and *korA*) (Figure 5). However, some minor differences observed were that pRAS4-F878/91 and pRAS4-F830/91 had *hin1*, which was not found in pYKCT010 and pYK0414. Also, *hin2* was not found in pYK0414 but it was present in pRAS4-F878/91, pRAS4-F830/91 and pYKCT010. Put together, all four plasmids shared a similar backbone structure consisting of genes associated with formation of a T4SS nanomachinery, replication, integration, and stable inheritance. Finally, the two pRAS4 plasmids (pRAS4-F878/91 and pRAS4-F830/91) encoded an operon that had two topoisomerase A (*topA*), eamA efflux pump, and Tet A efflux pump together with its Tet(R) A repressor that were not found in the Japanese pYKCT010 and pYK0414 plasmids (Figure 5).

## 4 Discussion

In this study, we present the first complete sequence of pRAS4 from *Aeromonas salmonicida* subsp. *salmonicida* strains F878/91 and F830/91, and demonstrate that pRAS4 is a functional self-transmissible conjugative plasmid that carries a novel replicon of environmental origin together with a complete set of canonical conjugation modules. The mating experiments produced antibiotic-resistant transconjugants in *Escherichia coli* DH5α (Section 3.3), and the antimicrobial susceptibility testing of the purified transconjugants confirmed selective transfer of the tetracycline resistance phenotype encoded by pRAS4 (Section 3.1). The conjugation machinery comprises a MobA/VirD2 family relaxase, the *mobC* accessory relaxosome gene, the canonical virB1–virB11 mating pair formation cluster, and a TraG/VirD4 family Type IV coupling protein (T4CP) that had been misannotated by Prokka as *traN* (Section 3.6). These findings provide a molecular explanation for the previously observed self-transmissibility of pRAS4 described by L’Abée-Lund and Sørum (2002), and provide the molecular basis for the self-transmissibility observed in that earlier study. The successful conjugation also indicates that pRAS4 has a host range extending beyond *Aeromonas*, since it is functional in an enteric recipient (*E. coli*) consistent with the broad host range character reported for many conjugative plasmids of Gram-negative bacteria (Norman and Hansen, 2009).

Our findings show that both pRAS4-878/91 and pRAS4-830/91 encoded an operon having 10 virB proteins that form a canonical T4SS nanomachinery consistent with gene clusters that form the MPF module in other bacteria species (Costa et al., 2021). In Gram negative bacteria, the virB proteins that form a T4SS nanotube are divided into four functional complexes that consist of (i) the extracellular pilus complex, (ii) outer membrane (OM) complex, (iii) inner membrane (IM) complex, and (iv) cytoplasmic ATP energy complex. VirB2/virB5 proteins that form the extracellular pilus complex were present in pRAS4. Of the two, virB2 serves as the major pilin protein while virB5 forms the outer pilin tip protein (Christie et al., 2014; Costa et al., 2015). The mating pair formation (MPF) cluster of pRAS4 comprises ten *virB* genes encoding the canonical components of a Gram-negative T4SS nanomachinery: virB1, virB2, virB3, virB4, virB5, virB6, virB8, virB9, virB10, and virB11 (Figure 4 A and 4B). Based on this canonical *virB1-virB11* organization, pRAS4 is classified within the MPF_T type (Guglielmini et al., 2014). The outer membrane channel by virB9 and virB10 (Christie and Cascales, 2005; Oliveira et al., 2016), the inner membrane subassembly by virB3, virB6 and virB8, and the cytoplasmic ATPase complex by virB4 and virB11 (Arechaga et al., 2008; Planet et al., 2001; Ripoll-Rozada et al., 2013; Rivas et al., 1997; Tato et al., *2005*). A 140 amino acid CDS in the operon was originally annotated by Prokka as virB7; however, InterProScan analysis returned no TrbC/VirB7 (Pfam PF03743) or other T4SS-component domain hit for this ORF at the default threshold (Section 3.6). This protein is therefore considered a small lipoprotein of uncertain function rather than a canonical virB7, consistent with the observation by Hofreuter et al. (2003) that virB7, is dispensable for plasmid DNA transfer in some T4SS nanomachineries.

In contrast to the conclusion of our initial Prokka-based analysis, pRAS4 does encode a Type IV coupling protein (T4CP). The 823-amino-acid ORF originally labelled *traN* was reassigned in this study, based on multi-database InterProScan analysis and BLASTp homology, as a TraG/VirD4 family T4CP (Section 3.6). The protein carries the canonical bipartite architecture of T4CPs of the TrwB/TraG/TraD/VirD4 family: two N-terminal transmembrane helices anchoring a cytoplasmic AAA+ ATPase domain with a Walker A motif (AAPTRSGKG) at residues 138–146 (Cabezón et al., 2015; Gomis-Rüth et al., 2001). T4CPs in the family couple relaxosomes to the secretion channel and provide energy for substrate translocation through the T4SS (Gonzalez-Rivera et al., 2016; Ripoll-Rozada et al., 2013). The identification of the pRAS4 T4CP, combined with the canonical MPF cluster, the MobA/VirD2 family relaxase and the *mobC* accessory relaxosome gene (Section 3.6), establishes that pRAS4 encodes the complete molecular system required for self-transmissible conjugation. The Prokka misassignment of this CDS as *traN* most likely reflects the propagation of similar mis-annotations through public sequence databases, as illustrated by the occurrence of TraG/VirD4 architecture proteins deposited under the name “TraN” in NCBI nr (e.g., CAC79168.1; Section 3.6). The functional demonstration of conjugation reported in Section 3.3 is consistent with this corrected annotation. Nevertheless, pRAS4-F878/91 and pRAS4-F830/91 share 91.19 and 91.14% nucleotide identity with pYKCT010 and pYK0414, respectively, indicating that these plasmids share a conserved synteny of genes that form their T4SS nanomachinery despite being from bacteria found in different aquatic environments and geographical areas.

Genes encoding additional mating pair formation accessory functions are also present in pRAS4. The *traO* gene encodes part of the core transmembrane subcomplex that functions as a cross-bridge channel enabling plasmid ssDNA transfer by spanning both the IM and OM complexes (Goessweiner-Mohr et al., 2014), and *trbM* contributes to effective transfer of plasmid ssDNA from donor to recipient cells (Gordils-Valentin et al., 2024). The presence of the entry exclusions gene *traS,* which prevents the transfer of plasmid ssDNA into cells that already carry the same plasmid (Kingsman and Willetts, 1978; Skurray et al., 1976; Ou, 1980), indicates that pRAS4 encodes regulatory genes that prevent excess plasmid DNA deposition in recipient cell as a means of preventing lethal zygosis. The high similarity in composition and organization of these accessory MPF genes between pRAS4-F878/91 and pRAS4-F830/91 from Norwegian *A. salmonicida* and the plasmids pYKCT010 and pYK0414 from uncultured bacteria in Japanese river sediments further consolidates the observation that all four plasmids share a conserved synteny of MPF accessory genes alongside their *virB1-virB11* cluster.

The relaxosome of pRAS4 comprises the MobA/VirD2 family relaxase identified in this study (Section 3.6) and the *mobC* accessory gene, which was originally annotated by Prokka as MobC domain-containing protein on the basis of BLAST similarity, although it did not return a significant InterProScan domain hit at the default threshold. MobC is a small relaxosome accessory protein that binds *oriT* and extends DNA strand separation at the nick site, facilitating relaxase-mediated cleavage of plasmids dsDNA to generate the ssDNA substrate that passes through the T4SS (Zhang and Meyer, 1997; Zhang, 1998). This presence of the single stranded DNA-binding protein (*ssB*) may protect the incoming ssDNA from degradation by host nucleases and facilitate its conversion to dsDNA in the recipient (Al Mamun et al., 2021; Demanèche et al., 2001). The replication initiator RepA is responsible for initiating replication of plasmid ssDNA after entry into the recipient cell (Chattoraj et al., 1985; Mott and Berger, 2007), and the partitioning genes *parB* and *soj* (a *parA* equivalent) form a Type I partitioning system that ensures stable segregation of replicated plasmids into transconjugants (Baxter and Funnell, 2014; Ebersbach and Gerdes, 2005; Williams and Thomas, 1992). The presence of *parB* and *soj* in pRAS4 is consistent with stable inheritance of the plasmid in transconjugant populations, ensuring that the entire population of pRAS4 recipients can act as potential donors in subsequent conjugation events (Boampong et al., 2020; Liang et al., 2014). The *korA* gene, whose product tightly regulates plasmid replication and stability by limiting the expression of conjugative transfer (*tra*) genes (Jagura-Burdzy and Thomas, 1994; Moré et al., 1996), is also present, indicating that pRAS4 encodes regulatory genes that modulate its own transfer. The high similarity in the composition and organization of these replication, relaxosome and maintenance components across pRAS4-F878/91, pRAS4-F830/91, pYKCT010 and pYK0414 supports the conclusion that all four plasmids share a conserved backbone.

Despite the presence of a complete and functional conjugation machinery similar to that of the canonical IncU plasmids, pRAS4 cannot be assigned to the IncU group based on sequence replicon typing. PlasmidFinder v2.0 at the 80% identity threshold returned no replicon match for pRAS4, and BLASTp analysis of the 453-amino-acid RepA protein returned no close match to the RepA proteins of the canonical IncU plasmids pRAS1, pAr-32 or pRA3 (Kulinska et al., 2008). Instead, the closest characterized RepA homologs of pRAS4 are found in environmental plasmids of *Acidithiobacillu* sp. (90.93% amino acid identity), unclassified *Acidovorax* (79.35%)*, Burkholderia stabilis* (80.82%), *Microvirgula aerodenitrificans* (70.26%), and *Massilia* sp. (65.65%). (Section 3.7), the same set of environmental β and γ proteobacteria that contain the closest homologs of the pRAS4 T4CP and relaxase. The co-occurrence of the closest homologs of three independent functional components the replication initiator, the relaxase and the coupling protein in the same set of environmental plasmids indicates that pRAS4 carries a previously uncharacterized plasmid backbone of aquatic environmental origin. Although L'Abée-Lund and Sørum (2000, 2001) and Sørum et al. (2003) classified pRAS1 and pAr-32 as IncU on the basis of classical incompatibility testing pRAS4 does not belong to any currently named IncU group based on sequence-based replicon typing. This is an important refinement of the molecular classification of the pRAS plasmid family.

The selective transfer of tetracycline resistance, but not sulphonamides or trimethoprim resistance from the *A. salmonicida* donor strains to *E. coli* DH5α (Section 3.1, Section 3.3) confirms that the tetracycline resistance phenotype of strains F878/91 and F830/91 is plasmid-encoded rather than chromosomal. Both donor strains showed the same resistance profile (tetracycline-resistant, sulphonamides and trimethoprim-susceptible) on disc diffusion and MIC testing, and the transconjugants inherited only the tetracycline resistance, with sulphonamides and trimethoprim susceptibility unchanged from the *E. coli* DH5α recipient. The transconjugant tetracycline MIC (16 μg/mL) matched that of the donors, consistent with transfer of the pRAS4 encoded Tet A efflux pump and repressor. Because pRAS4 is the only plasmid in the donor strains carrying a tetracycline-resistance determinant (Tet A) (Section 3.5, Table 5), and because *E. coli* DH5α carries no chromosomal tetracycline-resistance determinant, the tetracycline resistance acquired by the transconjugants is most parsimoniously transfer of pRAS4. Plasmid profiling demonstrated that a 50 kb plasmid was transferred to the recipient *E. coli* DH5α providing the transconjugant with phenotypic tetracycline resistance from a tetracycline resistant *A. salmonicida* subsp. *salmonicida* donor. Quantitative mating assays performed at the time of strain isolation in 1993 (Section 3.3, Table 3) showed that pRAS4 transfers to *E.coli* DH5α at frequencies as high as 2.88 × 10^−2^ transconjugants per recipient cell on agar (F878/91 donor) and 6.25 × 10^−2^ on agar (1993/91 donor), spanning approximately five orders of magnitude across replicate trials and dropping by one to four orders of magnitude in broth (Table 3). Frequencies of 10^−2^ per recipient cell are comparable to those reported for the canonical IncU plasmid pRAS1 and other broad-host-range self-transmissible R plasmids of Gram-negative bacteria, and are in order of magnitude higher than the transfer frequencies reported for the helper-dependent mobilization of pRAS3 by pRAS2 (L’Abée-Lund and Sørum, 2002; Sørum et al., 2003), establishing pRAS4 as a bona fide self-transmissible conjugative plasmid rather than a mobilization element. These quantitative results, together with the antimicrobial susceptibility data from the purified transconjugants (Section 3.1 and Section 3.2) and the agarose gel evidence of plasmid transfer (Figure 2), confirm that pRAS4 is a self-transmissible R-plasmid. The strong preference for surface-mediated mating, with agar frequencies one to four orders of magnitude higher than broth frequencies, is consistent with the use of an MPF_T-type VirB/VirD4 conjugation system, which assemblies a short rigid pilus that mediates efficient mating on solid surfaces but only ineffectively in liquid culture. The convergence of three independent lines of evidence, the quantitative transfer frequencies reported here, the recovery of single-colony-purified transconjugants in fresh mating experiments with selective tetracycline-resistance and confirmatory MIC testing (Section 3.1–3.3), and the InterProScan based identification of a complete MOB_P/MPF_T conjugation system including a TraG/VirD4 Type IV coupling protein and a MobA/VirD2 relaxase (Section 3.6) establishes that the conjugation machinery of pRAS4 is functional. The large trial-to-trial variability in transfer frequency (up to three orders of magnitude on agar) is typical of plasmid conjugation assays and is consistent with the sensitivity of the conjugation process to subtle differences in donor and recipient physiology, including the recently observed dependence of transfer on the use of fresh donor cells revived directly from −80 °C glycerol stocks (Section 3.3).

Our findings show that both pRAS4-F830/91 and pRAS4-F878/91 had an operon that carried the TetA efflux pump, TetR repressor and EamA efflux transporters linked to two *tnpA* that were all not present in pYKCT010 and pYK0414. TetA is an efflux pump tightly controlled by its TetR repressor that regulates tetracycline resistance even at low concentration levels (Orth et al., 2000; Saenger et al., 2000; Møller et al., 2016). As pointed out by Møller et al. (2016) that the TetA efflux pumps is so tightly controlled by TetR that it is responsive at sub-MIC tetracycline concentrations. It is likely that this tight regulation could have influenced the low MIC values of 16 μg/mL detected in *A. salmonicida* strains F878/91 and F830/91. The presence of the TetA efflux pump and its TetR repressor could account for tetracycline resistance seen in the disc diffusion test and the low MIC values of 16 μg/mL seen in *A. salmonicida* strains F878/91 and F830/91. The EamA efflux transporter is a member of the drug/metabolite transporter (DMT) superfamily, which has been linked to drug resistance as well as exporting drugs, metabolites and toxins from bacteria cells to the outside environment (Jack et al., 2001; Antony et al., 2019; Ahmad et al., 2026). The DMT superfamily transporters are crucial for the survival of bacteria in adverse environments, serving as a defense mechanism that pumps out toxin compounds from bacteria cells. Thus, the presence of the eamA efflux transporter gene in pRAS4-F830/91 and pRAS4-F878/91 could be an environmental adaptation acquired to enhance the survival of *A. salmonicida* by extruding toxin substances like drugs and metabolites (Konings, 2006; Yu et al., 2022). The presence of the *tnpA*, which is part of the Tn*3* transposable elements that play an important role in copying and pasting of different genes in bacteria genomes, indicates that the operon encoding the Tet A determinant and eamA efflux transporters was inserted into pRAS4 using the two *tnpA*. Overall, these findings suggest that transposases like *tnpA* can be used to copy and paste operons encoding efflux transporters that can be used as a defense mechanism by extruding toxin substances from bacteria cells.

The environmental origin of pRAS4 is supported by two independent lines of evidence. First, the closest BLASTp homologous of three independent pRAS4 proteins-the RepA replication initiator, the TraG/VirD4 Type IV coupling protein, and the MobA/VirD2 relaxase-all map to the same set of environmental β- and γ-proteobacterial plasmids of *Acidovorax, Microvirgula, Massilia, Acidithiobacillus* and *Burkholderia* (Sections 3.6 and 3.7). Second, the pRAS4 backbone shares extensive nucleotide-level synteny with the plasmids pYKCT010 and pYK0414 from uncultured bacteria of Japanese river sediments (Section 3.9). The convergence of these two independent observations indicates that pRAS4 belongs to a previously uncharacterized lineage of environmental plasmids of aquatic origin. The presence of tetracycline resistance in pRAS4 isolated in Norway may illustrate the very efficient modulation of the plasmid sequence by the Tn*3*-like transposon that became a result of the use of antibiotics to control furunculosis before effective vaccines were developed. The lack of close similar plasmids in GenBank except those two found from river sediments in Japan indicates that still pRAS4 is a rare environmental plasmid that with this study is revealed to be an insight into an early development of an R-plasmid. This underlines the potential global environmental link between aquatic bacteria in freshwater systems and the global risk of using antibiotics in open aquatic systems anywhere on the globe. This study also reminds everyone that using antibiotics to treat infectious diseases in aquaculture species is not a sustainable activity.

The presence of multiple plasmids in both *A. salmonicida* strains highlights a complex plasmid architecture consisting of conserved and structurally diverse elements. Small plasmids such as pAsa1 and pAsa2 appear highly stable and conserved across geographical and temporal scales, whereas other plasmids such as pAsa3 and pAsa5 were recovered as fragmented contigs in the genome assemblies. This is most likely attributable to limitations of short-read Illumina sequencing, particularly in resolving repetitive regions and structurally complex plasmids, rather than representing true biological variation (Wick et al., 2017). Such limitations are well documented in plasmid reconstruction using short-read sequencing technologies. The fragmented assembly of pAsal1 and pAsa5 in our study further underscores these technical limitations. The discrepancy between the cumulative contig sizes and the expected full-length reference sequences likely reflects incomplete assembly rather than true biological absence. Further resolution of these plasmids would require long-read or hybrid sequencing approaches.

## 5 Conclusion

In this study, we have identified the gene clusters that form the backbone modules of pRAS4. Comparative genomics analysis has revealed that pRAS4-F878/91 and pRAS4-F830/91 found in *A. salmonicida* subsp. *salmonicida* strains F878/91 and F830/91 isolated from Atlantic salmon in Norway in 1991 shares a conserved gene synteny that form a similar backbone structure with pYKCT010 and pYK0414, found in uncultured bacteria from river sediments in Japan in 2021. Mating experiments confirmed that pRAS4 is a functional self-transmissible conjugative plasmid, and domain-based re-annotation identified a TraG/VirD4 family Type IV coupling protein and a MobA/VirD2 family relaxase that had previously eluded annotation, completing the molecular complement of the pRAS4 conjugation machinery. Replication typing and RepA homology analysis further showed that pRAS4 carries a novel replicon that does not belong to any currently named Inc group. The four plasmids encode a similar backbone structure consisting of modules that regulate plasmid transfer, replication, maintenance and ensuring stable inheritance in recipient cells after transfer from donor cells. Put together, the high similarity in the composition and organization of these modules in the four plasmids points to an environmental lineage, which could have led to acquisition of analogous gene clusters in the plasmids by adapting to similar environmental challenges in different geographical areas. The high degree of conservation in the conjugation, replication, and maintenance modules of pRAS4 and the related environmental plasmids suggest that pRAS4 originated from a conjugative environmental plasmid backbone that subsequently acquired antimicrobial resistance determinants under antibiotic selection pressure.

Collectively, our findings indicate that *A. salmonicida* strains harbor a diverse plasmid repertoire comprising both conserved and structurally dynamic elements. Within this plasmid network, pRAS4 represents a conjugative backbone of environmental origin that has likely evolved into an R-plasmid under selective pressure. This study provides new insights into the evolutionary pathways of conjugative plasmids and emphasizes the role of aquatic environments as reservoirs of mobile genetic elements contributing to antimicrobial resistance dissemination.

## Data Availability

The complete plasmid sequences generated in this study have been submitted to the NCBI GenBank database under accession numbers PX997076 and PZ097580. The associated whole genome sequencing data are currently under embargo and will be made publicly available upon publication of this study.

## Footnotes

1
https://usegalaxy.no/

2
usegalaxy.org.au

3
https://www.ebi.ac.uk/interpro/

4
https://blast.ncbi.nlm.nih.gov/
